# Study on Joint Connection Performance of an Innovative Tooth Groove Connection and Vertical Reinforcement Lapping in Reserved Hole

**DOI:** 10.3390/ma16237371

**Published:** 2023-11-27

**Authors:** Xiaoyong Luo, Yang He, Qi Chen, Linsong Chen

**Affiliations:** 1 College of Civil Engineering, Central South University, Changsha 410075, China; csu-luoxy@csu.edu.cn (X.L.); tmgc1he@163.com (Y.H.); 18855440707@163.com (L.C.); 2 Engineering Technology Research Center for Prefabricated Construction Industrialization of Hunan Province, Changsha 410075, China

**Keywords:** joint connection performance, monotonic horizontal load test, tooth groove connection, vertical reinforcement lapping, precast concrete wall panel

## Abstract

In order to explore the horizontal joint connection performance of the innovative tooth groove connection and vertical reinforcement lapping in the reserved hole, five horizontal joint specimens were designed and constructed in this paper. Through the combination of monotonic horizontal load tests and finite element simulation analysis, the effects of axial compression ratio, vertical reinforcement connection degree, reserved hole type, mortar strength, and tooth groove depth on the horizontal joint connection performance of innovative tooth groove connections and vertical reinforcement lapping in reserved holes were comprehensively analyzed and discussed. The results indicated that the specimens were subjected to penetration failure at the tooth groove joint, but the vertical reinforcements and UHPC in reserved holes can effectively transfer the stress, ensuring satisfactory connection performance. With the increase in axial compression ratio and vertical reinforcement connection degree, the joint connection performance enhanced gradually, while the reserved hole type had little effect on the joint connection performance. In addition, it was found that increasing the mortar strength and the tooth groove depth can significantly improve the peak bearing capacity through finite element analysis. Finally, the optimization design suggestions for this innovative tooth groove connection and vertical reinforcement lapping in the reserved hole were given considering factors such as joint connection performance and construction assembly.

## 1. Introduction

As a modern construction engineering method, the precast structure prefabricates the building components in the factory and then assembles them on site. It has attracted much attention because of its significant advantages in improving construction efficiency, protecting the environment, and reducing construction time [1,2,3]. However, with the expansion of its application scope, the joint connection performance of the precast structure has attracted increasing attention [4,5,6]. Past research [4] has revealed that the joint connection in precast structures faces a multitude of challenges. Firstly, the joint connection needed to be installed quickly and accurately at the construction site; there were difficulties in quality control and engineering construction. Secondly, the joint connection is often needed to withstand complex loads and stress conditions, potentially leading to issues of material fatigue and fracture. Most significantly, the joint connection in precast structures had often not received adequate attention in terms of both design and construction, leading to structural problems such as detachment, deformation, or rupture [7,8,9]. These issues could pose threats to the overall stability of the building, making it susceptible to natural disasters like earthquakes. Joint connection, as a critical component of precast structures, demands a rational approach that not only facilitates effective transmission of internal forces within components, ensuring structural safety and reliability, but also aligns with the principles of industrialized production [10,11,12].

In recent years, novel joint connection methods for precast concrete structures have been emerging consistently. These methods primarily fell into two categories: wet connection and dry connection [13,14,15]. Scholars around the world have conducted extensive research on joint connection performance.

Hutchinson et al. [16] conducted monotonic horizontal load tests on nine full-scale precast concrete shear walls with post-tensioned horizontal connections. The test results indicated that the absence of bonded reinforcement reduced the lateral residual deformation of the precast wall panels, demonstrating excellent horizontal connection performance and load-bearing capacity. Research by Holden et al. [17], as well as Smith and Kurama et al. [18], suggested that the use of unbonded post-tensioning (UPT) came with higher construction costs and provided limited enhancements in the energy dissipation capacity of the wall panels. Fu et al. [19] proposed a new joint connection that involved vertical reinforcement lap welding and steel plate welding in the concealed columns at the edge of precast shear walls. Subsequently, the connection of the wall panel structures was achieved through bolting. When the test specimen reached its peak load, the welded steel plate connections had not yet yielded, indicating the rationality and reliability of this connection method. Zhao et al. [20] conducted a seismic performance evaluation of precast concrete wall panel structures with bolted connections. They also proposed a simplified restoring force model, providing valuable insights for the further application of bolted connections in precast wall panel structures. The majority of conventional dry connections suffered from a notable drawback: the connection elements were directly embedded in the concrete wall panels. This arrangement resulted in stress concentration in the connection elements, ultimately leading to extensive concrete spalling. Sun et al. [21] introduced an innovative high-strength bolt connection for precast wall panels. By utilizing high-strength bolts and H-shaped connectors, they transformed the connection between concrete elements into a steel-to-steel connection. Nevertheless, it was worth noting that this joint connection required complex assembly procedures, challenges in quality control during construction, and elevated maintenance costs, all of which hindered the widespread adoption of precast concrete structures.

Liao et al. [22] introduced an innovative joint connection of precast concrete shear walls, consisting of central precast wall panels and cast-in-place boundary components. The distributed reinforcement in the precast wall panels was disconnected at the joint, and the vertical reinforcement of the cast-in-place components was connected to ensure the bearing capacity of the shear wall. However, it was important to note that this design still needed cast-in-place components, which required on-site construction. Jia et al. [23] conducted a study on the hysteresis performance of precast frame shear wall structures connected with grouted sleeves. The experimental results demonstrated that this connection exhibited excellent energy dissipation capabilities and ductility, effectively transmitting reinforcement stresses. Focusing on the problems of the high cost and difficult processing of grouting sleeves, Gao et al. [24] introduced a new method of forming ribs on the inner wall of a sleeve by using surfacing welding. This joint connection method was convenient, minimized steel waste, and enabled parameterized control and automated production, making it suitable for a wide range of applications in practice. However, sleeve connections imposed high precision requirements on the installation of precast components, and their larger diameter made this connection method unsuitable for thin-walled precast wall panels. Gu et al. [25] introduced a novel precast shear wall with rebar lapping in grout-filled constrained hole connections. This innovative design allowed for effective horizontal joint connection through the spiral hoops and grout-filled holes. The test results demonstrated that the precast shear wall with this horizontal joint connection exhibited excellent structural performance. Zhi et al. [26] proposed a connection method involving lap splicing in grout-filled reserved holes. Experimental results from quasi-static tests indicated that this approach effectively transferred forces at the joint. Zhang et al. [27] investigated the steel anchorage performance and shear resistance of vertical grout anchoring connections in precast wall panels with different reinforcement ratios. The test results demonstrated that the anchoring of connection reinforcement, along with the interface between new and old concrete, could effectively connect the upper and lower wall panels. Apparently, grout splicing could overcome challenges related to high construction precision and the presence of extensive wet construction.

In summary, the existing connection methods have been proven through experiments for their effectiveness and reliability. However, for the grouting sleeve connection, the joint connection performance is greatly affected by the construction quality, including the uncontrollable grouting and the high precision assembly. The vertical grout anchoring connection has low requirements for assembly accuracy, but there are still challenges such as uncontrollable grout quality and easy bending of reinforcements due to the long lap length. Dry connection methods such as bolt connection and welded connection can greatly reduce wet construction, but they also involve difficulties with high construction accuracy, high assembly precision, and difficult maintenance in the future. These challenges hinder the realization of industrialized construction in precast concrete structures, which limits the application and development of such structures in the future [28,29].

In response to the challenges mentioned above and drawing from the research of Gu, Zhi, and Zhang et al. [25,26,27], our research group [30] previously introduced an innovative precast concrete structure system with tooth groove connection and vertical reinforcement lapping in the reserved hole, which is only composed of two components: a precast wall and a precast slab. And the horizontal joints are connected with tooth groove connections and vertical reinforcement lapping in the reserved hole, while the vertical joints are connected by the extended horizontal reinforcement lapping in the reserved concave tooth cavity and pouring self-compacting concrete. Taking a standard layout student apartment as the prototype structure, a three-story substructure was designed and manufactured using the above innovative connection method for the shaking table test. The results indicated that the substructure showed excellent deformation capacity and seismic performance, but the horizontal joints were relatively weak and were subjected to serious damage. Consequently, in order to further explore the horizontal joint connection performance of the innovative tooth groove connection and vertical reinforcement lapping in reserved holes and study the influence of axial compression ratio, vertical reinforcement connection degree, reserved hole type, mortar strength, and tooth groove depth on the horizontal joint connection performance, five horizontal joint specimens were designed and manufactured in this paper. A series of monotonic horizontal load tests and finite element analysis were carried out, and the optimization design suggestions of this innovative tooth groove connection and vertical reinforcement lapping in the reserved hole were given. It is hoped that the research results of this paper can promote the popularization and application of precast wall panels with this innovative horizontal joint connection.

## 2. Materials and Methods

### 2.1. Connection Technology

**Figure 1:**
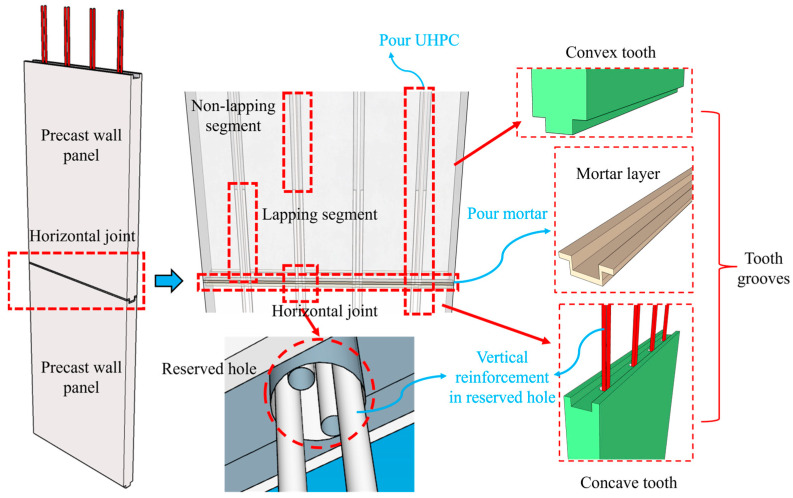
Connection details.

In order to solve the problems of high construction accuracy, the need for on-site formwork, uncompacted grouting, and difficulty in positioning and assembling existing horizontal joint connection methods, our research group proposed an innovative tooth groove connection and vertical reinforcement lapping in the reserved hole [30]. The connection details are shown in Figure 1.

As shown in Figure 1, there are convex and concave teeth at the top and bottom of the precast wall panels. The convex tooth and concave tooth are interlocked with each other and effectively connected as a whole through high-performance mortar. In addition, full-length holes are reserved on each precast concrete wall panel, and the vertical reinforcements are indirectly lapped in reserved holes poured by ultra-high performance concrete (UHPC) to ensure reliable bonding of the vertical reinforcements and effective stress transfer at the horizontal joint. The most prominent innovative advantage of this connection method is the tooth groove structure, which consists of a convex tooth and a concave tooth and is similar to the traditional mortise-tenon joint. This tooth groove structure can play an important role in rapid positioning in the assembly process, which greatly improves assembly efficiency on site. Moreover, the impermeability effect of the tooth groove joint is obviously better than that of the straight joint, which is also an obvious advantage. In addition, the use of vertical reinforcement lapping in the reserved hole greatly reduces the difficulty of on-site construction and has low requirements on the assembly accuracy of the components. And the reserved hole can be used as grouting templates during the assembly process to achieve on-site construction without the need for templates. The assembly can be completed only by simply pouring mortar at the tooth groove and grouting UHPC in the reserved hole, which greatly improves the on-site assembly efficiency and reduces the construction requirements for workers’ operations. In addition, UHPC also shows excellent properties such as extraordinary strength, high density, high durability, self-compacting, and a superior bond with reinforcement. This method significantly reduces the required length of steel overlapping compared to traditional vertical grout anchoring and ensures grouting quality in the construction [31,32], making it a reliable high-performance material for filling the vertical reinforcement lapping segments. Overall, the innovative tooth groove connection and vertical reinforcement lapping in the reserved hole show many advantages compared with other connection methods, and their connection performance is reliable.

### 2.2. Design of Specimen

**Figure 2:**
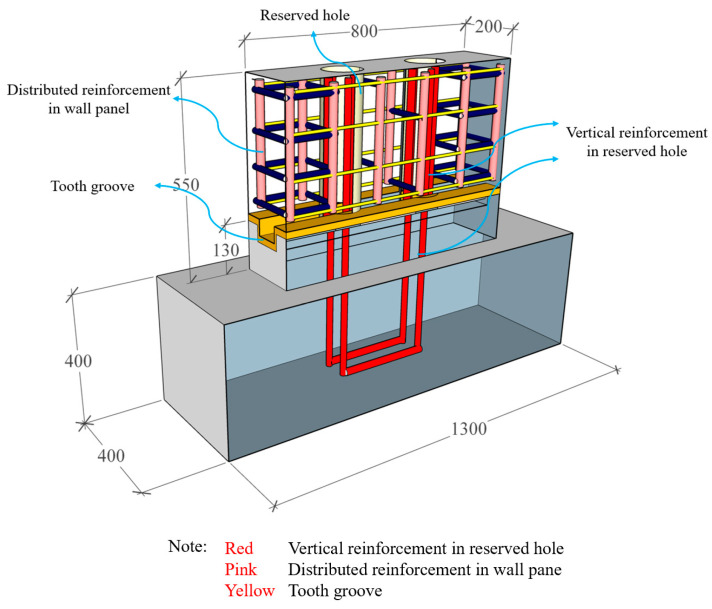
Specimen size diagram (Unit: mm).

In order to explore the variation law of the joint connection performance of the precast wall panel and further optimize the structural design of the horizontal joint connection, five precast wall panel specimens with tooth groove connections and vertical reinforcement lapping in the reserved hole were designed and fabricated in this test. The dimensions of the specimens are consistent at 800 mm × 550 mm × 200 mm, and the shear span ratio, defined as the distance from the loading point to the top of the basement divided by the length of the specimen, is set at 0.5. The size diagram of the specimen is shown in Figure 2.

**Figure 3:**
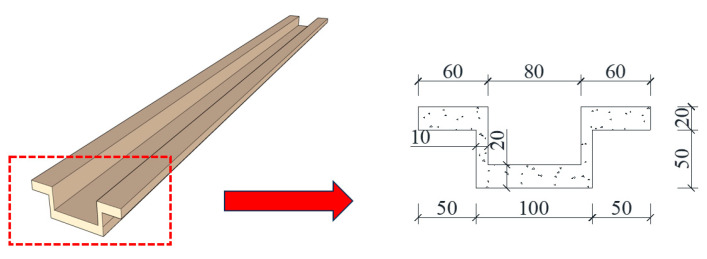
The tooth groove details (Unit: mm).

As shown in Figure 3, the tooth groove was higher on both sides and lower in the middle. Considering the effective bonding at the tooth groove, the upper and lower wall panels were connected as a unified entity with a 20 mm-thick mortar, which should be designed with a strength not lower than that of the concrete at the precast wall panel.

The monotonic horizontal load tests were conducted to investigate the influence of axial compression ratio, vertical reinforcement connection degree, and reserved hole types on the novel joint connection performance. The axial compression ratio *λ* refers to the ratio of the axial pressure on the specimen *N* to the product of the cross-sectional area *A* and the design value of the axial compressive strength of the concrete *f*_c_. The expression is shown in Equation (1).

*λ* = *N*/*f_c_·A* (1)

The axial pressure is to simulate the vertical load of the specimen in the actual structure. Precast wall panel with tooth groove connection and vertical reinforcement lapping in the reserved hole is suitable for multi-story building construction, which is in a low axial pressure state. The most unfavorable axial compression ratio is close to 0.1 at the bottom story according to the actual structural load calculation, and the axial pressure can be calculated (*N* = 0.1 × 14.3 × 200 × 800 = 229 kN). Considering the limitation of the range of the laboratory loading device, the joint connection performance of the top (0 axial compression ratio) and bottom (0.1 axial compression ratio) wall panel specimens is mainly studied.

**Figure 4:**
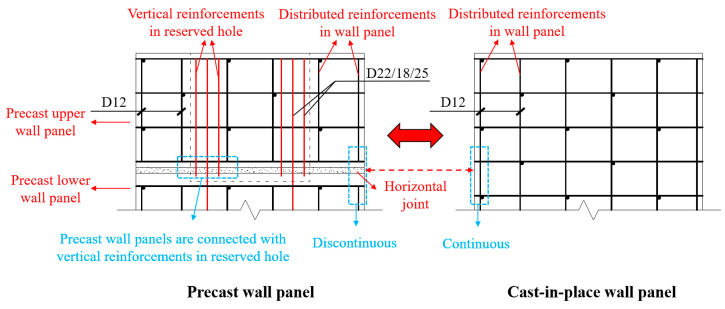
Comparison of cast-in-place and precast wall panels (Unit: mm).

As shown in Figure 4, compared with the cast-in-place wall panels, the distributed reinforcements in the precast wall panels are discontinuous at the joint and are only connected by the vertical reinforcements in reserved holes. Therefore, the degree of vertical reinforcement connection is defined as the ratio of the cross-sectional area of the vertical reinforcements in the reserved hole to the cross-sectional area of the distributed reinforcements. For instance, in the case of WP01, the vertical reinforcements in the reserved hole are configured at 4D22 (1520 mm^2^), while the distributed reinforcements in the cast-in-place wall panel are set at 12D12 (1357 mm^2^), resulting in a reinforcement connection degree of approximately 100%, where “D” represents the symbol for steel reinforcement. The reinforcement ratio to the cross-section of precast concrete wall panel is 0.85% by calculation, which meets the requirements of the current standard [33]. The key parameters of the experimental design are shown in Table 1.

**Figure 5:**
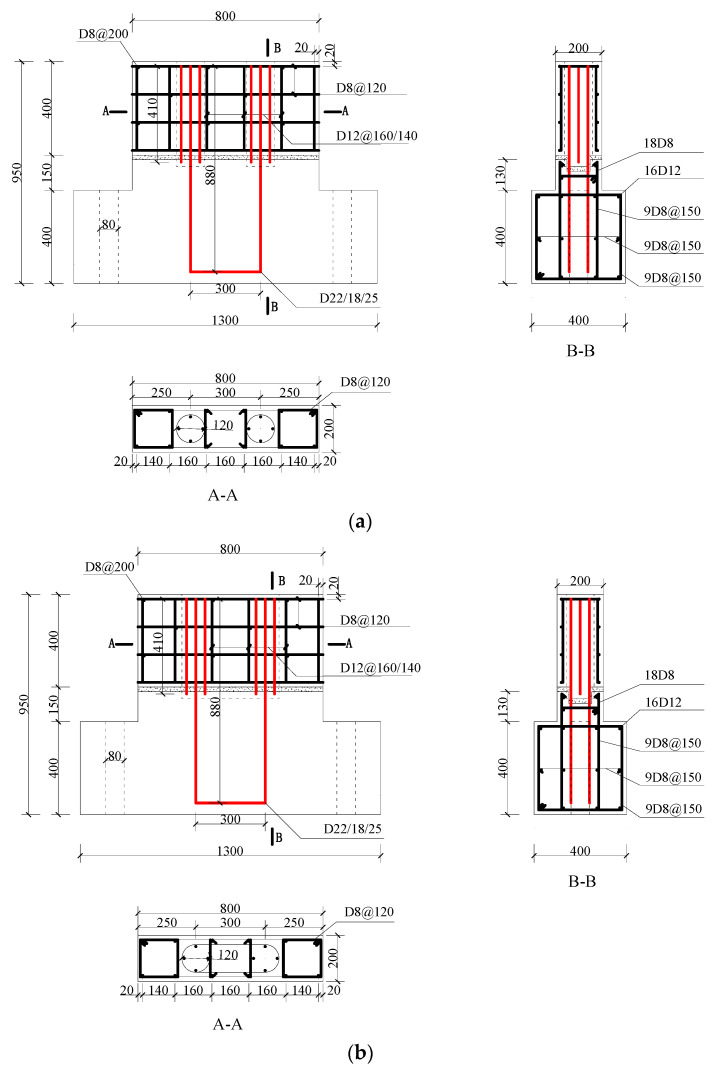
The reinforcement details of the specimens (Unit: mm): (**a**) WP01 (WP02, WP03, WP04); (**b**) WP05.

The precast wall panel specimens consisted of precast wall panels, precast basement, a mortar layer, and UHPC. The distributed reinforcements in the wall panel were arranged in a double-layer pattern and interconnected using tied reinforcements to form a unified entity. At the horizontal joint, the distributed reinforcement in the wall panel was discontinuous and not connected to the basement. In the basement, two U-shaped reinforcements were pre-embedded at the corresponding positions of the reserved hole, extending 400 mm above the concave tooth groove surface of the basement. These U-shaped reinforcements were of the same specifications as the vertical reinforcement inserted into the reserved hole of the precast wall panel, and they indirectly lapped together in the reserved hole. The distributed reinforcement in the wall panel has a diameter of 12 mm. Meanwhile, the vertical reinforcement in the reserved hole has diameters of 18 mm, 22 mm, and 25 mm, respectively. UHPC was poured in the reserved hole to realize the effective connection of the vertical reinforcement in the reserved hole. The reinforcement details of the wall panel specimens are shown in Figure 5. Due to the use of UHPC in the reserved hole grouting material, the lap length of vertical reinforcement in the reserved hole could be reduced to 12.6d [32]. For convenience of construction, a lap length of 400 mm was used.

### 2.3. Fabrication and Assembly

**Figure 6:**
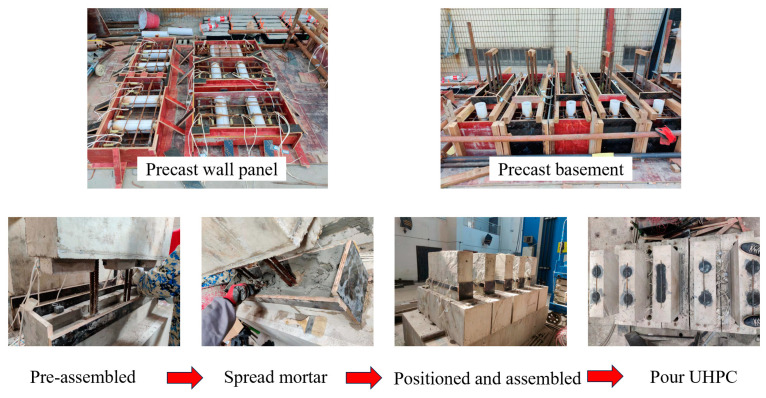
The assembly process of the specimens.

The precast basement and wall panel were poured separately. The full-length reserved holes of the wall panel were made of PVC pipe, and the U-shaped vertical reinforcements were pre-embedded in the basement. During the assembly process of the wall panel specimens, the “calibrate first, then level” principle was followed. First, the basement was positioned and leveled, and mortar was spread in the tooth groove. Then, the wall panel was positioned and assembled, ensuring that the pre-embedded reinforcements were inserted into their corresponding reserved holes. The vertical reinforcement was then inserted, evenly distributed in the reserved holes. Finally, UHPC was poured into the reserved holes to achieve effective bonding of the vertical reinforcement. After curing with water for 28 days, the specimens were prepared for a monotonic horizontal load test. The assembly process of the specimens is shown in Figure 6.

### 2.4. Material Properties

The concrete strength of the wall panel and basement is C30, the reinforcement is HRB400, and the mechanical properties of the reinforcements are shown in Table 2, where the elongation of the reinforcements *δ* refers to the ratio of the increased length after the tensile fracture of the reinforcement ∆*L* to the original length *L*. The expression is shown in Equation (2).

*δ* = ∆*L*/*L* (2)

Three concrete cube specimens were prepared for each test specimen to conduct compressive strength tests. The mortar used at the joint and the UHPC filled in the reserved holes were mixed on-site. According to the standard [34,35,36], the compressive strength test was carried out after the specimen was cured and formed. The test results are shown in Table 3.

### 2.5. Test Procedure

**Figure 7:**
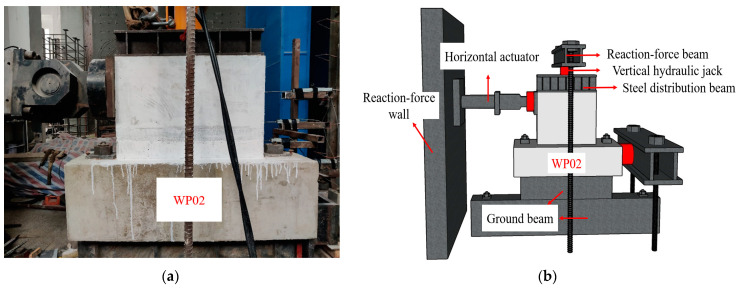
Test loading device: (**a**) Physical diagram; (**b**) Model diagram.

The schematic diagram of the test loading device is shown in Figure 7. The monotonic horizontal load test involves both vertical and horizontal loads. Initially, a vertical axial load was applied to the midsection of the steel distribution beam using a vertical hydraulic jack. The steel distribution beam evenly distributed the load to the top of the specimen, maintaining a constant vertical load throughout the test to simulate the vertical load on the specimen. Simultaneously, a horizontal load was applied to the top of the specimen using a horizontal actuator. Load–displacement control was employed, where the test initially followed a load control with increments of 10 kN. Once the wall panel specimen yielded, it was switched to displacement control. The test was terminated when the horizontal load dropped to 85% of the peak load or the specimen was damaged.

**Figure 8:**
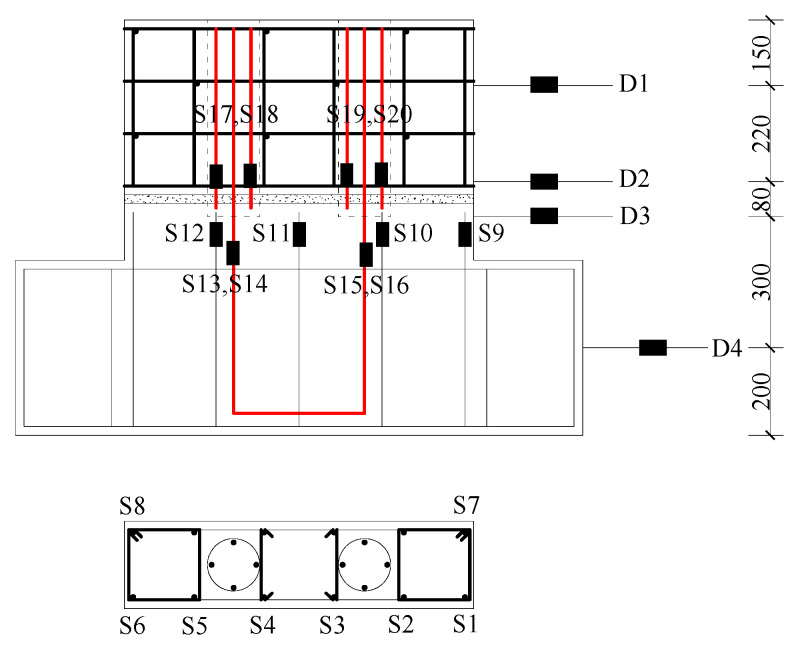
Measuring points layout (Unit: mm).

As shown in Figure 8, on one side of the specimen, there were a total of four displacement sensors arranged as follows: D1 measured the horizontal displacement of the wall panel at the same height as the horizontal actuator. D2 and D3 measured the relative displacement of the wall panel on both sides of the horizontal joint, indicating the lateral displacement in the joint. D4 measured the sliding displacement in the basement. It can be seen from Figure 8 that a total of 20 steel strain measuring points were arranged in the specimen, of which S1–S12 were the distributed reinforcement steel strain gauges and S13–S20 were vertical reinforcement steel strain gauges, which were distributed at both ends of the lap section.

## 3. Results and Discussion

### 3.1. Test Phenomena and Failure Modes

Through monotonic horizontal load tests, all specimens exhibited a consistent structural failure pattern, which could be conducted in four stages: crack initiation and development, yield, peak, and ultimate stages.

**Figure 9:**
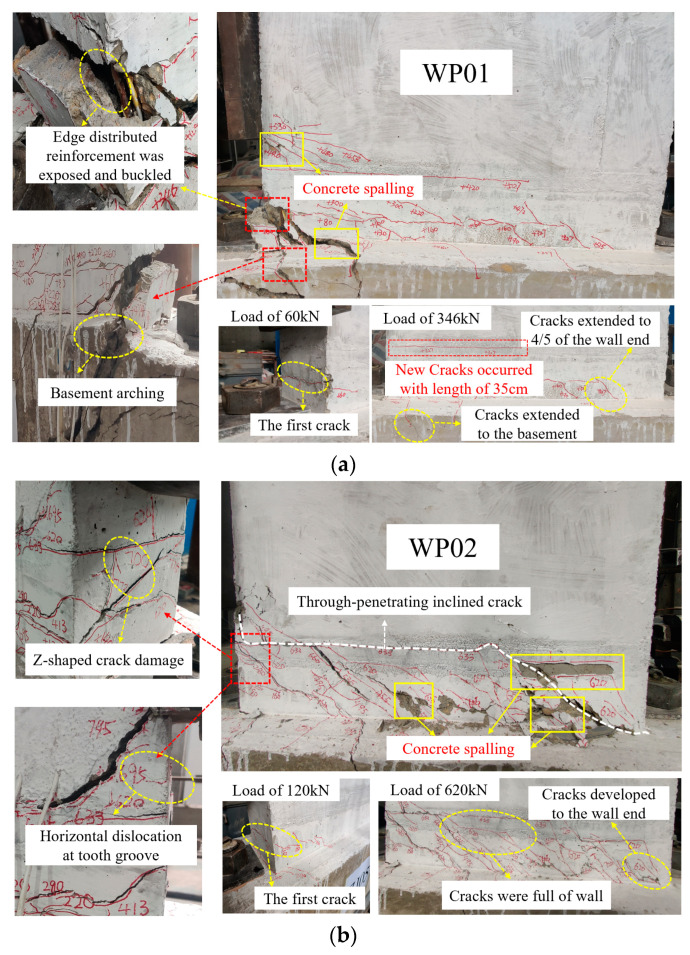

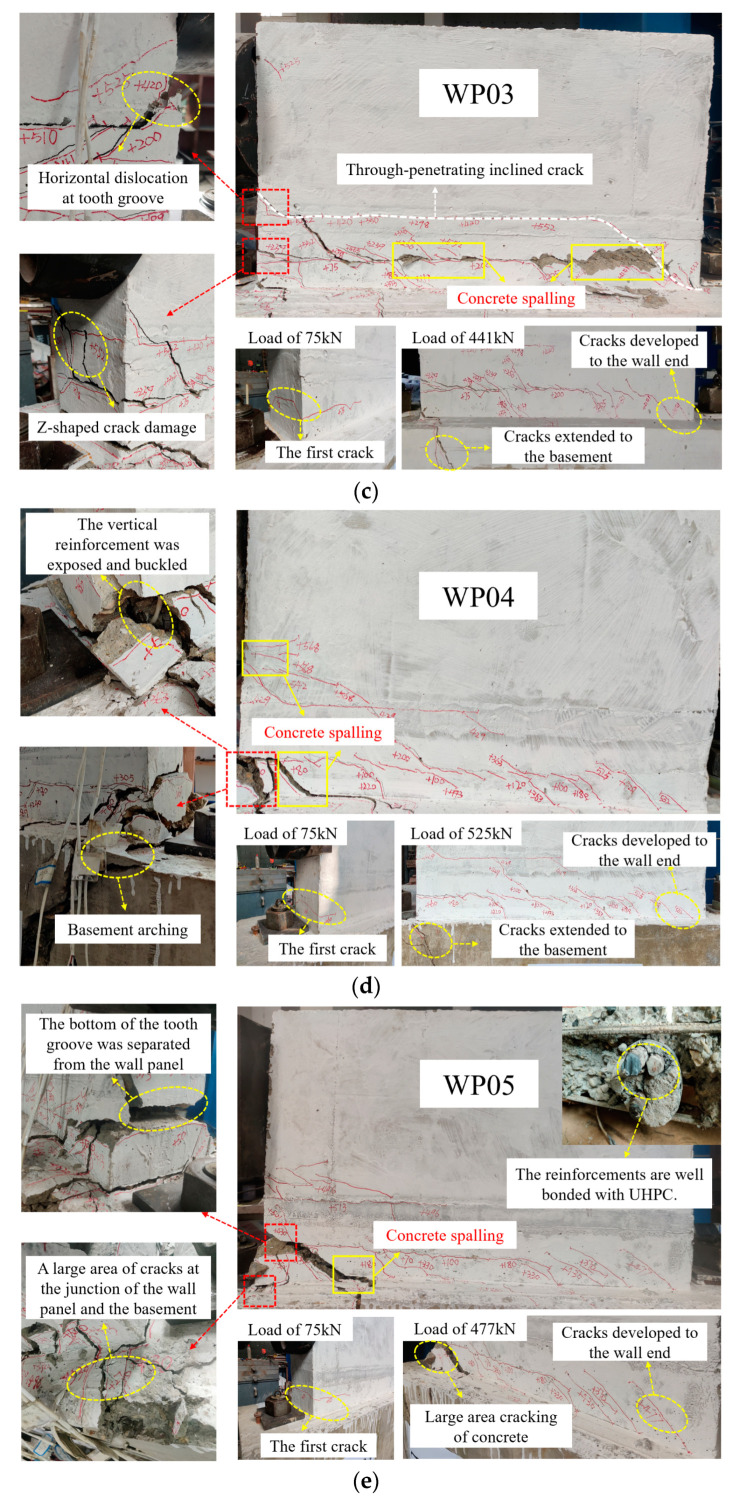
Failure modes of specimens: (**a**) WP01; (**b**) WP02; (**c**) WP03; (**d**) WP04; (**e**) WP05.

During the crack initiation and development stages, each specimen initially exhibited an elastic state with minimal residual deformation. Horizontal cracks first appeared at the bottom of the tooth groove, closer to the horizontal actuator side, and gradually extended to the surface of the wall panel. During the yield stage, the deflection of all the specimens increased nonlinearly. Horizontal cracks at the bottom of the tooth groove developed diagonally away from the horizontal actuator side, accompanied by 45-degree inclined cracks extending to the basement. During the peak stage, the cracks at the bottom of the tooth groove and at the junction of the wall panel and the basement near the horizontal actuator side widened significantly, and concrete exhibited tensile failure and spalling, while concrete on the side further from the horizontal actuator underwent compression failure. During the ultimate stage, both the upper and lower surfaces of the tooth groove exhibited prominent cracking, edge-distributed reinforcements became exposed and buckled, and the central portion of the basement exhibited conspicuous arching. However, it was noteworthy that specimens WP02 and WP03 exhibited more extensive crack development during the ultimate stage, including through-penetrating inclined cracks and horizontal dislocation at the tooth groove, with a displacement of approximately 2 mm. The failure modes of the specimen are shown in Figure 9.

The comparative analysis of the failure modes of the specimens is as follows:

Comparing the crack propagation in specimens WP01 (0 axial compression ratio) and WP02 (0.1 axial compression ratio) with 100% vertical reinforcement connection degree, it is revealed that the axial compression ratio has a significant impact on crack development. Specimen WP02 exhibited more extensive crack propagation, with cracks uniformly distributed along the wall panel. This phenomenon arises from the fact that the existence of axial compression causes an increase in the angle between the principal tensile stress direction at various points of the specimen and the axis of the specimen in comparison to specimens with a 0 axial compression ratio. Consequently, the angle between the critical inclined cracks and the axis of the specimen becomes smaller. This results in an expanded shear compression area in the wall, ultimately enhancing the overall load-bearing performance of the wall panel. In general, the axis compression ratio has a significant influence on the failure mode of the precast wall panel with tooth groove connection and vertical reinforcement lapping in the reserved hole.

In a comparison of Figure 9a,c,d, it is evident that the crack propagation in specimens WP01 (100% vertical reinforcement connection degree), WP03 (67% vertical reinforcement connection degree), and WP04 (134% vertical reinforcement connection degree) with a circular reserved hole is nearly identical. While crack propagation in the specimen WP03 was more extensive, the distribution of cracks along the tooth groove direction was relatively uniform. There were through-penetrating inclined cracks with horizontal dislocation in the tooth groove section, and at the end of the wall, there was Z-shaped crack damage. In contrast, specimens WP01 and WP04 exhibited a failure mode characterized by concrete tensile spalling near the actuator end, with no horizontal dislocation in the tooth groove section. In general, the vertical reinforcement connection degree of 100% and 134% is excessive, while the 67% vertical reinforcement connection degree allows for more extensive crack development and better load-bearing performance of the wall panel.

Comparing Figure 9a,e, it can be seen that the crack propagation of specimens WP01 (reserved circular hole) and WP05 (reserved rounded rectangle hole) with 100% vertical reinforcement connection degree is basically similar, and the crack initiation position and development direction on the wall panel are roughly the same. Moreover, the inclined cracks extended to the junction of the wall panel and the basement, and the concrete spalling near the actuator side was more obvious. In general, the failure mode and concrete spalling degree of specimens WP01 and WP05 are basically consistent, indicating that the reserved hole type has no significant effect on the failure mode of the precast wall panel with tooth groove connection and vertical reinforcement lapping in the reserved hole.

### 3.2. Load–Displacement Curve

**Figure 10:**
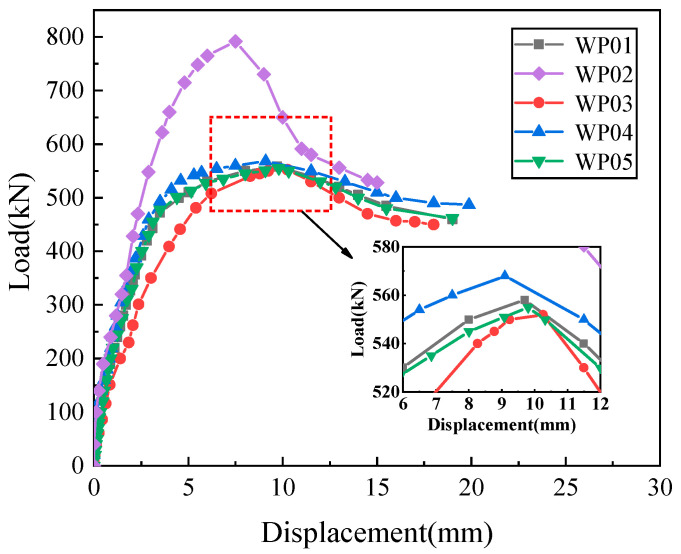
Load–displacement curves.

A comparison of the load–displacement curves for the five specimens carried out with a monotonic horizontal load is illustrated in Figure 10. Table 4 provides the characteristic load values for each stage of the specimens. The analysis of the test results is as follows:

When the vertical reinforcement connection degree and reserved hole type are consistent among the specimens, it is evident that the peak bearing capacity of the specimen with the axial compression ratio of 0.1 is significantly higher than that of the specimen with the axial compression ratio of 0. The reason is that the existence of an axial compression ratio not only inhibits the initiation and propagation of inclined cracks and increases the friction force at the horizontal joint interface, resulting in the specimen being in a two-way stress state, but also gradually increases the angle between the main tensile stress direction and the axial direction, which in turn increases the height of the concrete shear zone, thus enhancing the bearing capacity of the precast wall panel. It is indicated that the existence of axial compression can significantly improve the joint connection performance of the precast wall panel.

When the axial compression ratio on the specimen and the reserved hole type are consistent, the load–displacement curves for the three grades of vertical reinforcement connection degree remain largely consistent. It can be observed that as the degree of vertical reinforcement connection increases, there is a slight increase in both the degree of peak load and the ultimate load of the specimens. This suggests that the vertical reinforcement connection between 67% and 134% has a minimal impact on the joint connection performance of the precast wall panels.

When the axial compression ratio and vertical reinforcement connection degree are consistent among the specimens, it is observed that the load–displacement curves of the circular hole and rounded rectangle hole specimens are essentially identical, indicating that the reserved hole type has a minimal impact on the joint connection performance of the wall panels. This is because the joint connection performance is mainly determined by the interface bonding force and friction force in the tooth groove area and vertical reinforcements lapping in the reserved hole. The reserved hole is mainly for pouring grouting material to ensure effective bonding of vertical reinforcements and effective stress transfer at the horizontal joint. Consequently, for the reinforcement and structural design of various practical engineering projects, the appropriate reserved hole type can be selected.

Table 4 lists the crack load *F_cr_*, crack displacement ∆*_cr_*, yield load *F_y_*, yield displacement ∆*_y_*, peak load *F_p_*, peak displacement ∆*_p_*, ultimate load *F_u_*, ultimate displacement ∆*_u_*, horizontal dislocation displacement ∆*_h_* and displacement ductility coefficient *μ*. According to Table 4, it is evident that the axial compression ratio has a significant impact on the load-bearing capacity of the specimens. The existence of the axial compression significantly increases the peak load-bearing capacity of the specimen by 41.9%, and the horizontal dislocation displacement of the joint is decreased. On the other hand, the vertical reinforcement connection degree on the peak load-bearing capacity of the joint is relatively small in the range of 67–134%. As the connection degree increases from 67% to 134%, the peak load-bearing capacity only experiences a modest increase of 1.8%. Additionally, the reserved hole type has almost no effect on the peak load-bearing capacity of the specimens.

### 3.3. Ductility

As the main anti-lateral force component in the building structure, the ductility of the precast wall panels plays an equally important role in the joint connection performance [37,38]. Table 4 lists the displacement ductility coefficient *μ* to quantify the deformation capacity of the precast wall panels. The expression is shown in Equation (3).

*μ* = ∆*_u_*/∆*_y_* (3)

This joint connection method has great deformation ability compared with the traditional joint [19,21,22,23]. The displacement ductility coefficient of the wall panels proposed in this paper is greater than 3, which is due to the large displacement of the wall panels caused by the dislocation of the specimen at the joint, and the vertical reinforcement in the reserved hole has a certain dowel action. It can be seen from Table 4 that the existence of an axial compression ratio can significantly reduce the ductility of the specimen [39]. This is because under the same displacement, the compressive stress of the specimen with a large axial compression ratio is greater, so the deformation capacity of WP02 is lower than that of other specimens. It is worth noting that the ductility of the specimen increases with the increase in the vertical reinforcement connection degree, which indicates that enhancing the diameter of the vertical reinforcement in the reserved hole can improve the deformation ability of the specimen. Therefore, a precast wall panel with a tooth groove connection and vertical reinforcement lapping in the reserved hole has good ductility performance.

### 3.4. Steel Strain

**Figure 11:**
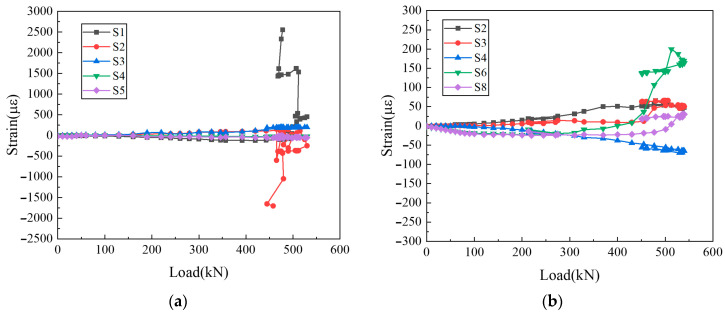
Distributed reinforcement strains of wall panel: (**a**) WP01; (**b**) WP05.

**Figure 12:**
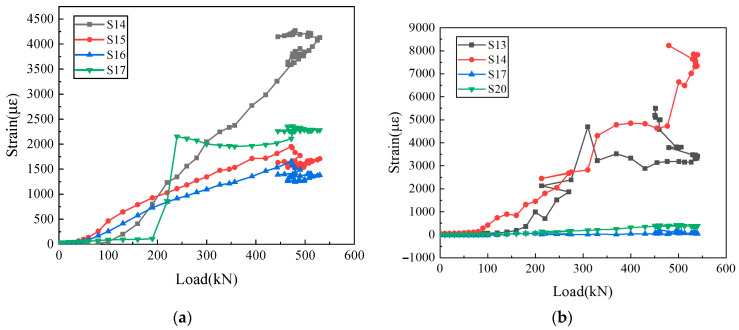
Vertical reinforcement strains in the reserved hole: (**a**) WP01; (**b**) WP05.

The strain–load curves for the distributed reinforcement of the wall panel and the vertical reinforcement in the reserved hole are depicted in Figure 11 and Figure 12.

It is evident from Figure 11 that, before the failure of the specimen, the distributed reinforcement strain was approximately linear with the applied load. Notably, the distributed reinforcement strain at the edge of the wall panel is higher than that of the distributed reinforcement strain in the middle, although both remain below 500 με. The corner measurement points S1 and S2 exhibit a tendency of tensile and compressive strain during the failure phase, which is due to the dowel action of the vertical reinforcement in the reserved hole to suppress the horizontal sliding between the upper and lower wall panels. Consequently, the upper wall panels uplift to transfer stress. From the overall point of view, the distributed reinforcements of the precast wall panel mainly play a structural role.

It can be indicated from Figure 11 and Figure 12 that during the yield stage, the distributed reinforcement strains in the wall panel remain below 500 με. In contrast, the lower vertical reinforcement in the reserved hole lapping segment exhibits strains approaching 1500 με, which is higher than that at the upper end. It can be seen from the test phenomenon that the failure of the horizontal joint area precedes the overall failure of the precast wall panel. This shows that in this innovative type of joint connection, the vertical reinforcements in the reserved hole make a great contribution to bearing the load. Additionally, UHPC is poured into the reserved hole to facilitate robust bonding with the vertical reinforcements and ensure efficient stress transfer at the horizontal joint, leading to outstanding joint connection performance for the precast wall panel.

## 4. Simulation

### 4.1. Finite Element (FE) Modeling

**Figure 13:**
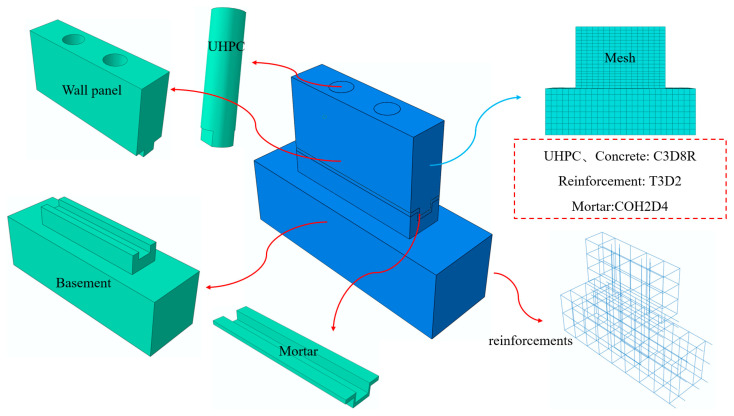
Finite element modeling process.

To further investigate the joint connection performance of the new precast wall panels, finite element analysis was conducted using the software ABAQUS 6.14. The finite element modeling process is illustrated in Figure 13. Concrete and UHPC materials were simulated using the concrete plastic damage model in ABAQUS [40], which calculated the tensile–compressive stress–strain relationship and the tensile–compressive damage–strain relationship of concrete based on standard [41]. The material properties of the reinforcements were modeled using a bilinear model, including both the elastic and strengthening stages. Based on material testing, the average values for the yield strength and ultimate strength of the reinforcements were 450 N/mm^2^ and 630 N/mm^2^. For the strengthening stage, a modulus of elasticity of 0.01Es was used, where Es is the elastic modulus. In the model, the concrete and the UHPC in the lapping segment were all represented using solid elements (C3D8R), while the reinforcements were modeled using truss elements (T3D2), and the mortar layer adopted the cohesion element (COH2D4). The distributed reinforcements were embedded in the concrete, and since the vertical reinforcements in the reserved hole bonding with UHPC were well-anchored during the experiments, they were also embedded in the UHPC. The contact between the reserved hole and the UHPC was modeled using tie constraints. This finite element analysis allows for further investigation of the joint connection performance of the precast wall panels under a monotonic horizontal load.

### 4.2. Model Verification

**Figure 14:**
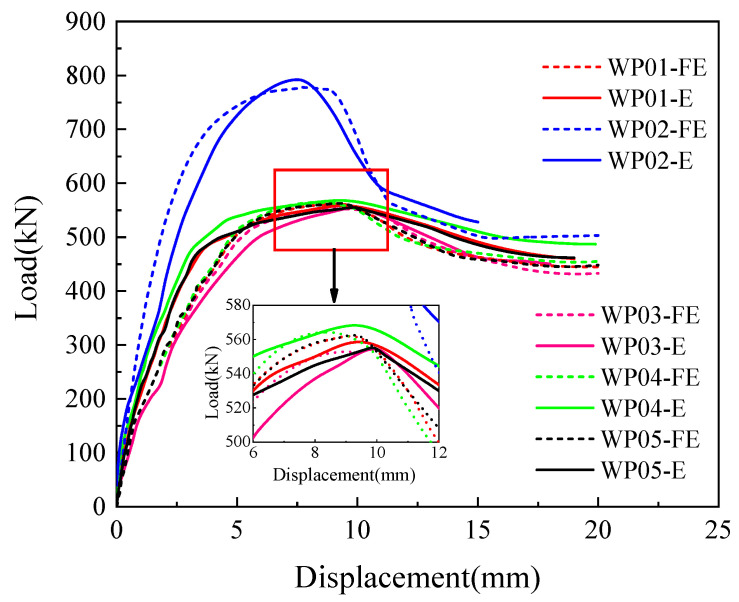
Load–displacement curve comparison of finite element analysis results and the experimental results.

The finite element model established above was used to simulate the crack development and failure behavior of the specimens under a monotonic horizontal load in the tests. And the finite element analysis results and the experimental results of the load–displacement curve are shown in Figure 14, where FE represents finite element analysis results and E represents experimental results.

It can be seen from Figure 14 that the finite element analysis is in close agreement with the experimental results. The load–displacement curves all exhibited distinct stages, characterized by elastic, strengthening, and descending stages. Notably, the experimental results displayed the bond failure and slip of the reinforcements during the testing process. In contrast, the finite element model employed an “embedded” contact approach to simulate the interaction between reinforcements and concrete without considering the effect of bond slip between concrete and reinforcements. Consequently, the numerical model exhibited a marginally earlier peak displacement compared to the experimental data. However, when viewed comprehensively, this model effectively approximates the joint connection performance of precast wall panels with tooth groove connections and vertical reinforcement lapping in reserved holes.

**Figure 15:**
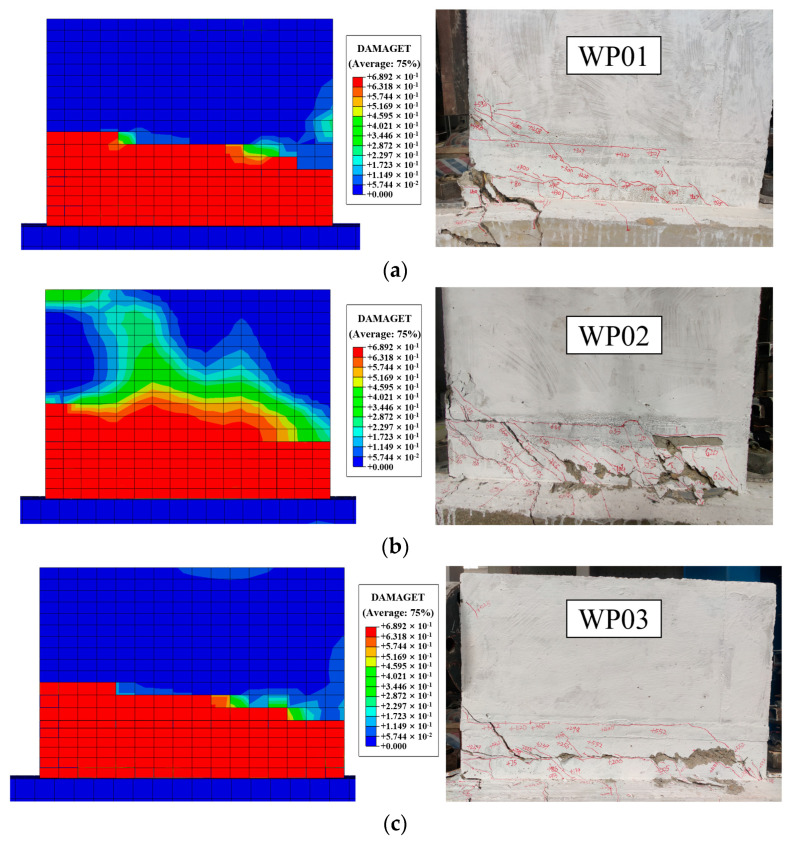

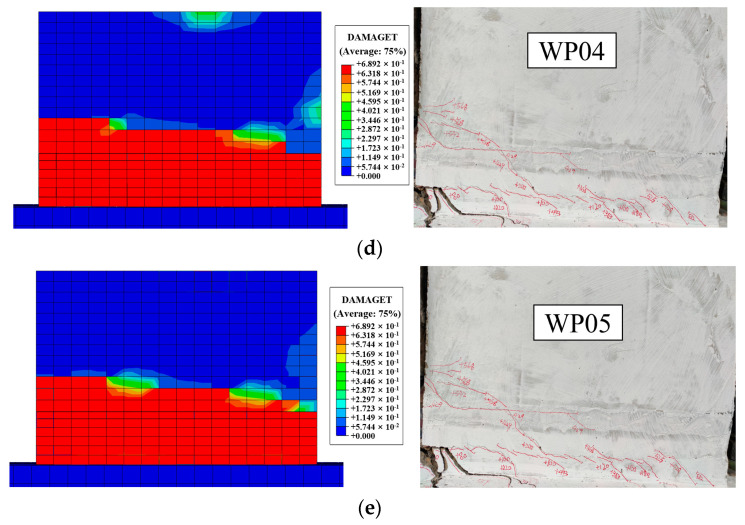
Failure modes comparison of finite element analysis results and the experimental results: (**a**) WP01; (**b**) WP02; (**c**) WP03; (**d**) WP04; (**e**) WP05.

The distribution of cracks in the wall panel primarily depends on the tensile damage of the concrete, as indicated by the DAMAGET [42], which depicts the damage distribution of concrete in the process of tension. A higher value of DAMAGET represents a greater level of damage occurring in the concrete. The simulated tensile damage profile of the specimens is depicted in Figure 15. In the numerical simulation process, the first horizontal crack appeared at the bottom of the tooth groove. With the increase in load, the crack gradually extended to the other end of the specimens and developed diagonally to the junction of the wall panel and the basement, eventually forming a through-penetrating inclined crack. The tooth groove experienced horizontal penetration failure during the test process, which was basically consistent with the failure mode obtained by the above simulation. It can be concluded that the simulation of tensile damage closely matched the actual crack distribution observed in the experiment.

The characteristic values of experimental results and finite element analysis results are compared in Table 5, where *F_y_* and *F_p_* represent the yield load and peak load of the specimen during the experimental results, and *F_y,s_* and *F_p,s_* represent the yield load and peak load obtained from the load–displacement curve of finite element analysis results.

It is evident from Table 5 that the finite element simulation results are very similar to the experimental results, and the variation coefficients of yield load and peak load are 0.04 and 0.01, respectively. Therefore, the finite element constitutive model employed in this study accurately simulates the actual mechanical behavior of the specimens during monotonic horizontal load tests. It can be effectively applied to the parameter analysis of precast wall panels with tooth groove connections and vertical reinforcement lapping in reserved holes.

### 4.3. Parametric Analysis

#### 4.3.1. Axial Compression Ratio

**Figure 16:**
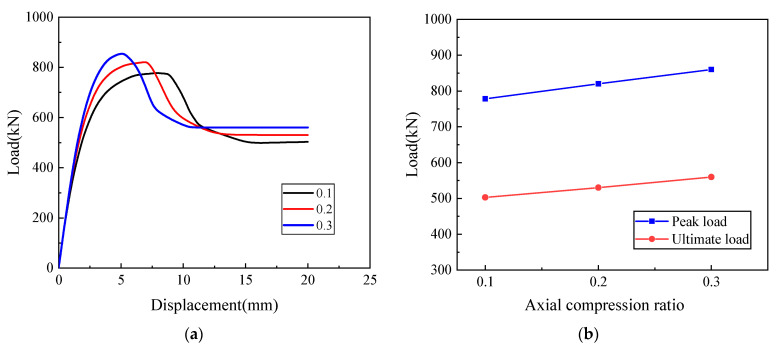
The influence of axial compression ratio: (**a**) Load–displacement curve; (**b**) Peak load and ultimate load curves with axial compression ratio.

To investigate the influence of axial compression ratio on the joint connection performance of precast wall panel with tooth groove connection and vertical reinforcement lapping in reserved holes, the finite element model of the WP02 specimen with a vertical reinforcement connection degree of 100% and reserved circular holes was taken as an example to compare and analyze the variation law of joint connection performance of precast wall panel specimens with axial compression ratios of 0.1, 0.2, and 0.3. Based on the finite element analysis results, the load–displacement curves of the specimens under different axial compression ratios are plotted, as shown in Figure 16.

It is evident from Figure 16 that as the axial compression ratio increases, the initial stiffness and peak load of the specimens slightly increase while the ductility decreases. After reaching the peak load, the load–displacement curve becomes steeper, and the length of the plastic zone significantly decreases. This indicates that an axial load ratio between 0.1 and 0.3 can significantly enhance the bearing capacity of precast wall panel specimens. However, it simultaneously reduces the deformation capacity of the specimens. The reason is that the existence of an axial compression ratio not only inhibits the initiation and propagation of inclined cracks and increases the friction force at the horizontal joint interface, resulting in the specimen being in a two-way stress state, but also gradually increases the angle between the main tensile stress direction and the axial direction, which in turn increases the height of the concrete shear zone, thus enhancing the bearing capacity of the precast wall panel with tooth groove connection and vertical reinforcement lapping in the reserved hole.

#### 4.3.2. Vertical Reinforcement Connection Degree

**Figure 17:**
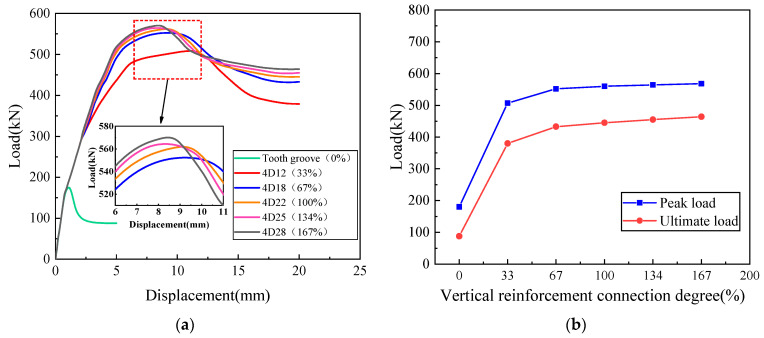
The influence of vertical reinforcement connection degree: (**a**) Load–displacement curve; (**b**) Peak load and ultimate load curves with vertical reinforcement connection degree.

Based on the monotonic horizontal load test results of the precast wall panel with tooth groove connection and vertical reinforcement lapping in reserved holes, the effect of the bearing capacity of the precast wall panel with vertical reinforcement connection degree was further studied. Based on the parameters of the WP01 specimen, the variation law of joint connection performance of specimens with different vertical reinforcement connection degrees of 0%, 33%, 67%, 100%, 134%, and 167% was analyzed, respectively. The results of finite element analysis are depicted in Figure 17.

It can be seen from Figure 17 that the specimen with a 0% vertical reinforcement connection degree shows relatively weak joint connection performance and obvious brittle characteristics. The distributed reinforcements in the wall panel are discontinuous at the joints, and the joint connection performance only depends on the interface bonding force and friction force. In the absence of axial compression, the friction force is relatively small, resulting in the obvious brittleness of the specimen during failure. In contrast, improving the vertical reinforcement connection degree can significantly enhance the joint connection performance of precast wall panels. This highlights the substantial contribution of vertical reinforcement in the reserved hole to resisting loads in monotonic horizontal load tests. When the vertical reinforcement connection degree increases from 33% to 67%, the bearing capacity of the specimen increases by 8.9%, while the bearing capacity of the specimen is not significantly improved when the vertical reinforcement connection degree is greater than 100%. Therefore, for this new type of tooth groove connection and vertical reinforcement lapping in the reserved hole, the degree of vertical reinforcement connection in the reserved hole should be properly balanced to avoid insufficient or wasteful reinforcement. Overall, it is suggested that the appropriate vertical reinforcement connection degree should be 67–100%.

#### 4.3.3. Mortar Strength

**Figure 18:**
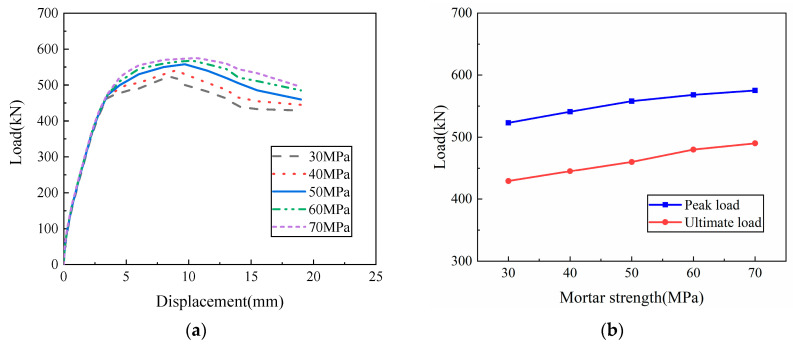
The influence of mortar strength: (**a**) Load–displacement curve; (**b**) Peak load and ultimate load curves with mortar strength.

In order to explore the influence of mortar strength on the joint connection performance of the precast wall panel with tooth groove connection and vertical reinforcement lapping in the reserved hole, based on the parameters of the WP01 specimen, five grades of mortar strength of 30, 40, 50, 60, and 70 MPa were selected for comparative analysis. The simulation results are depicted in Figure 18.

It can be seen from Figure 18 that the joint connection performance of the specimens increases with the increase in mortar strength. When the mortar strength increases from 30 MPa to 70 MPa, the peak load-bearing capacity of the specimen increases by approximately 10%. The reason is that the joint connection of the tooth groove mainly depends on the interaction between the mortar and the interface of the precast concrete. Similar to the interface of the old and new concrete, the high-strength mortar has high strength, dry shrinkage, and can fill the surface voids of the precast concrete. Therefore, the bonding force between the interface of the precast concrete and the mortar can be greatly improved, leading to enhanced joint connection performance of the precast wall panel with tooth groove connection and vertical reinforcement lapping in the reserved hole. In practical engineering, the mortar strength at the horizontal joints of the precast wall panel with tooth groove connection and vertical reinforcement lapping in the reserved hole can be selected as needed, but not less than 30 MPa.

#### 4.3.4. Tooth Groove Depth

**Figure 19:**
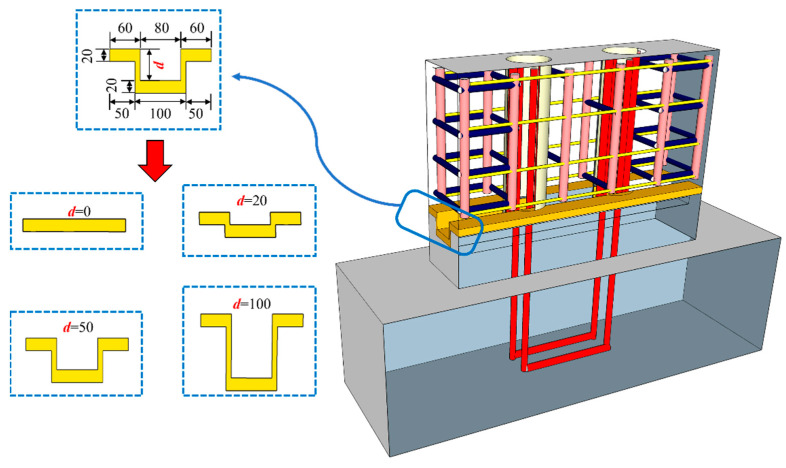
Local detail diagram of tooth groove with different depths (Unit: mm).

**Figure 20:**
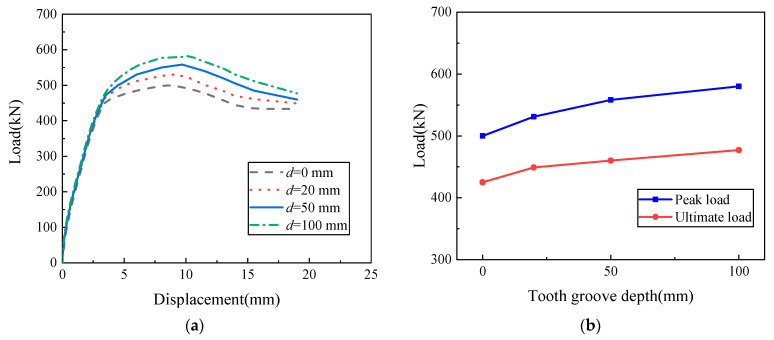
The influence of tooth groove depth: (**a**) Load–displacement curve; (**b**) Peak load and ultimate load curves with tooth groove depth.

The local detail diagrams of different tooth groove depths are shown in Figure 19 (d represents the tooth groove depth in Figure 19). The total width of the tooth groove is the same as the wall thickness, which is 200 mm. Based on the parameters of WP01, the peak bearing capacity of model specimens with four different tooth groove depths (0 mm, 20 mm, 50 mm, and 100 mm) was studied. The simulation results are illustrated in Figure 20.

It can be seen from Figure 20a that all specimens show similar initial stiffness. When the peak load is reached, the curve decreases and gradually stabilizes at a certain value. In addition, Figure 20b shows that the bearing capacity of the specimen increases with an increase in tooth groove depth. Compared with the specimen with a tooth groove depth of 0 mm, the peak bearing capacity of the specimen with a tooth groove depth of 50 mm increased by 11.6%. The reason is that enhancing the depth of the tooth groove can increase the friction area of the horizontal connection interface to strengthen the interlocking and friction actions at the horizontal joint, which improves the joint connection performance of the precast wall panel with tooth groove connection and vertical reinforcement lapping in the reserved hole. However, it is worth noting that the excessive tooth groove depth will cause it to be easily damaged during hoisting and transportation. Therefore, the recommended optimal tooth groove depth of the precast wall panel with tooth groove connection and vertical reinforcement lapping in the reserved hole is 50–100 mm.

## 5. Conclusions

In this paper, an innovative tooth groove connection and vertical reinforcement lapping in reserved holes method were introduced, and their joint connection performance was explored by monotonic horizontal load tests and finite element analysis. The conclusions are as follows:

(1) The typical failure mode of the specimens with a tooth groove connection and vertical reinforcement lapping in the reserved hole was the horizontal through-penetrating inclined crack at the tooth groove connection. The distributed reinforcements mainly served a structural role, and the effective stress transfer at the horizontal joint was mainly achieved through the tooth groove interlocking action and the vertical reinforcements and UHPC in reserved holes. This innovative tooth groove connection and vertical reinforcement lapping in reserved hole method showed excellent connection performance, with the advantages of simple structure, convenient positioning, no template, and efficient assembly.
(2) With the increase in the axial compression ratio and vertical reinforcement connection degree, the joint connection performance of the specimens with tooth groove connection and vertical reinforcement lapping in the reserved hole significantly increased. However, when the vertical reinforcement connection degree was too large, the improvement of the joint connection performance was not obvious, and excessive reinforcement would cause waste. It was suggested that the reasonable value of the vertical reinforcement connection degree was 67–100%. In addition, the joint connection performance of the specimens with the reserved circular hole and the rounded rectangle hole was not significantly different and could be selected according to construction needs.
(3) The finite element model of the specimens with tooth groove connection and vertical reinforcement lapping in the reserved hole was established, and its accuracy was verified. Through parameter analysis, it was found that with the increase in mortar strength and tooth groove depth, the joint connection performance improved. The reason was that increasing the mortar strength can improve the bond behavior between the interface of the precast concrete and the mortar, while increasing the tooth groove depth can strengthen the interlocking and friction actions. Considering the issues of hoisting, assembly, and transportation, it was recommended that the reasonable tooth groove depth be 50–100 mm.
(4) In this paper, UHPC with ultra-high mechanical properties and durability was explicitly designated as the grouting material in reserved holes, which come with certain limitations. However, grouting materials with different compressive strengths and fluidities would affect the anchoring effect and grouting compactness of the lapping section. Therefore, the effect of different grouting materials on joint connection performance should be further studied in the future.

## Figures and Tables

**Table 1:** Specimen parameter.

Specimen	Reserved Hole Type	Axial Compression Ratio	Vertical Reinforcement Connection Degree
WP01	Circular hole	0	4D22 (100%)
WP02	Circular hole	0.1	4D22 (100%)
WP03	Circular hole	0	4D18 (67%)
WP04	Circular hole	0	4D25 (134%)
WP05	Rounded rectangle hole	0	4D22 (100%)

**Table 2:** The mechanical properties of reinforcements.

Specification	Yield Strength *f_y_*/Mpa	Ultimate Strength *f_u_*/Mpa	Elongation *δ*/%
D8	453.6	635.0	20.4
D12	435.4	553.0	21.6
D18	447.7	625.3	28.4
D22	447.9	628.1	28.4
D25	465.8	655.6	26.3

**Table 3:** Compressive strength of concrete, mortar, and UHPC.

Materials	Dimension/mm	Load/kN	Compressive Strength *f_c_*/Mpa
Mortar	70.7 × 70.7 × 70.7	165	50.5
UHPC	100 × 100 × 100	1398	139.8
Concrete	150 × 150 × 150	648	28.8

**Table 4:** The characteristic load values for each stage of the specimens.

Specimen	*F_cr_*/kN	∆*_cr_*/mm	*F_y_*/kN	∆*_y_*/mm	*F_p_*/kN	∆*_p_*/mm	*F_u_*/kN	∆*_u_*/mm	∆*_h_*/mm	*μ*
WP01	60	0.12	472	3.68	558	9.71	461	19.00	/	5.16
WP02	120	0.19	675	4.25	792	7.50	528	14.40	1.8	3.39
WP03	75	0.36	441	4.57	552	10.26	448	17.00	2.6	3.72
WP04	60	0.07	493	3.60	568	9.08	480	19.88	/	5.52
WP05	50	0.16	477	3.65	555	9.82	462	19.91	/	5.45

**Table 5:** Characteristic value comparison of experimental results and finite element analysis results.

Specimen	*F_y_*/kN	*F_y,s_*/kN	*F_p_*/kN	*F_p,s_*/kN	*F_y,s_*/*F_y_*	*F_p,s_*/*F_p_*
WP01	472	461	558	560	0.98	1
WP02	675	713	792	778	1.06	0.98
WP03	441	455	552	555	1.03	1.01
WP04	493	470	568	564	0.95	0.99
WP05	477	463	555	558	0.97	1.01
Average value					0.998	0.998
Coefficient of variation					0.04	0.01

## Data Availability

All the data obtained from this study are already given in the article.
